# A new oral liquid formula including high dose prednisolone

**DOI:** 10.1186/2045-7022-1-S1-P107

**Published:** 2011-08-12

**Authors:** Patra Staubach, Adriane Groffik, Evgenij Goloborodko, Heidrun Mitzel-Kaoukhov, Joachim Saloga

**Affiliations:** 1 Department of Dermatology University Mainz, Mainz, Germany

## Background

Corticosteroids are active agents, which are especially applicated orally in the dermatological emergency medicine. Due to German anaphylaxis guidelines patients emergency kit must contain a corticosteroid with a Prednisolone-equivalent of at least 100 mg. Due to different reasons, a liquid corticosteroid is to be recommended. At the present time, there is no comparable liquid remedy available. The alternative is intravenously administered prednisolone. Due to this reason, a new formula is created by a research group of pharmacologists and dermatologists. They are working close to the Pharmaceutical Laboratory of the New Prescription Formulatory (NRF) in Germany, which is developing new formulations, also the Prednisolone formula. The new formula contains a Prednisolone-equivalent up to 500 milligrams per 100 milliliters liquid agent.

## Methods

We studied 60 patients, who came on an emergency basis to our university hospital with severe acute or chronic spontaneous urticaria and/or angioedema, half of them including throat swallowing. We observed the efficacy of the new prednisolone liquid in different concentrations as well as possible adverse events during the course of the treatment in comparison to intravenously administered prednisolone.

## Results

Up to the dosage over 250 mg Prednisolone-equivalent – similar to the intravenous therapy with the same dosage – a fast reduction of the symptoms (less than 30 minutes) was realized in urticaria, angioedema and throat swallowing comparable with the intravenous injections. No adverse events occured.

## Conclusions

This new Prednisolone formula is a new therapeutic alternative rescue medication.

